# Knowledge, attitude, and practice of stroke and thrombectomy among medical students in Henan, China

**DOI:** 10.1097/MD.0000000000040441

**Published:** 2024-11-08

**Authors:** Haobo Gao, Pengcheng Zhu, Hongtu Tan, Lingfeng Shu, Qinghai Dai, Jiabin Wang, Tao Wu

**Affiliations:** a Department of Intervention, Encephalopathy Center, the First Affiliated Hospital of Henan University of Chinese Medicine, Zhengzhou, China.

**Keywords:** attitude, knowledge, practice, stroke, thrombectomy

## Abstract

To assess knowledge, attitude, and practice (KAP) of stroke and thrombectomy among medical students in Henan, China. A cross-sectional study was conducted on medical students from 5 universities in Henan, China between June and September, 2022, using a self-administered questionnaire. A total of 1105 medical students [697 (62.1%) females] participated. Their mean KAP scores were 11.1 ± 2.90, 35.37 ± 4.94, and 21.48 ± 5.51 out of 14, 44, and 24, respectively. Structural equation modeling revealed that, age (estimate = 0.351, *P* = .005) and education (estimate = 0.370, *P* = .024) positively affected knowledge, while major (estimate = −0.128, *P* = .017), internship experience in neurosurgery or neurology (estimate = −1.321, *P* < .001), and family history of stroke (estimate = −0.557, *P* < .001) negatively influenced knowledge. Knowledge (estimate = 0.649, *P* < .001) and having seniors over the age of 60 at home (estimate = 1.228, *P* = .001) had positive effects on attitudes, while internship experience in neurosurgery or neurology (estimate = −0.471, *P* = .090) and family history of stroke (estimate = −0.596, *P* = .020) had negative impact on attitudes. Moreover, knowledge (estimate = 0.230, *P* < .001) and attitudes (estimate = 0.628, *P* < .001) positively influenced practices, whereas sex (estimate = −1.141, *P* < .001), internship experience in neurosurgery or neurology (estimate = −0.578, *P* = .025), and family history of stroke (estimate = −0.523, *P* = .027) negatively influenced practices. Medical students in Henan, China showed adequate knowledge, positive attitude, and proactive practice toward stroke and thrombectomy. Age, sex, education, major, internship experience in neurosurgery or neurology, family history of stroke, having seniors over the age of 60 at home might have impact on their KAP.

## 1. Introduction

Stroke is a leading cause of death globally and the primary cause of adult disability in China,^[1]^ driven by risk factors such as hypertension, diabetes, smoking, and coronary heart disease factors.^[2]^ The Global Burden of Disease study reported a substantial increase in stroke cases and deaths between 1990 and 2019,^[3]^ with China facing a particularly high burden. In 2020, an estimated 3.4 million new stroke cases and 2.3 million stroke-related deaths occurred in China, highlighting the urgent need for stroke-related research and improved management strategies.^[4,5]^ Ischemic strokes, caused by vascular occlusion, account for 85% of cases, while the rest are due to intracerebral hemorrhage.^[6]^ Although intravenous thrombolysis is commonly used for acute ischemic stroke, its effectiveness is limited by a narrow 4.5-hour treatment window and several contraindications.^[7]^ Mechanical thrombectomy has emerged as a promising treatment for large artery occlusions, significantly improving outcomes within 6 to 24 hours of stroke onset.^[8]^ While widely adopted in Western countries, thrombectomy remains underutilized in China.^[9]^

The lag in adoption of thrombectomy in China can be partially attributed to a lack of awareness and understanding among healthcare providers. A recent study conducted in Shaanxi province found that healthcare workers scored an average of 19.56/23 in knowledge of ischemic stroke, and only 57.9% reported always recommending patients for thrombolysis or thrombectomy.^[10]^ Awareness of modern stroke interventions is crucial for early recognition and timely management, which are key factors in reducing stroke-related disability and mortality.^[11]^ Knowledge and attitudes toward stroke treatment play a significant role in shaping the practice of future healthcare professionals. However, there is a limited understanding of how well medical students, who represent the next generation of healthcare providers, are equipped with the necessary knowledge and practical skills to manage stroke, particularly regarding modern interventions such as thrombectomy.

This study aims to assess the knowledge, attitude, and practice (KAP) toward stroke and thrombectomy among medical students in Henan, China. As medical students are pivotal to the future of healthcare, evaluating their awareness and understanding of stroke interventions is critical to identifying educational gaps and shaping curricula that emphasize early recognition and treatment. By improving the KAP of medical students, the potential for enhanced stroke management practices and better patient outcomes can be realized, ultimately contributing to the reduction of stroke-related mortality and disability in China.

## 2. Methods

### 2.1. Study design and participants

This cross-sectional study was performed on medical students in Henan, China between October 2022 and January 2023. Participants were selected from 5 universities, including Zhengzhou University, Henan University, Xinxiang Medical College, Henan University of Science and Technology, and Henan University of Chinese Medicine, using a clustered sampling method. This study was approved by the Ethics Committee of our Hospital (2022HL-401). All participants were informed about the study protocol and provided written informed consent to participate in the study. I confirm that all methods were performed in accordance with the relevant guidelines. All procedures were performed in accordance with the ethical standards laid down in the 1964 Declaration of Helsinki and its later amendments.

## 3. Procedures

The questionnaire was designed in Chinese according to Chinese Stroke Association guidelines for clinical management of cerebrovascular disorders: executive summary and 2019 update of clinical management of spontaneous subarachnoid hemorrhage^[12]^ and modified following the suggestions of 6 specialists. The questionnaire was pretested in 30 medical students. The Cronbach’s alpha of overall scores was 0.894. Kaiser- Meyer-Olkin was 0.935.

The questionnaire was composed of 4 categories, including personal information, knowledge, attitude, and practice (Appendix 1, Supplemental Digital Content, http://links.lww.com/MD/N874). Knowledge score was assessed by 14 questions using a 2-point scale (1 = correct response, 0 = incorrect answer or do not know). The total knowledge score was between 0 and 14. The attitude was evaluated by 11 items. Except for the 7^th^ item, each item was assessed using a 5-point Likert scale ranging from 0 to 4 (0 = strongly disagree, 1 = disagree, 2 = neither agree nor disagree/do not know, 3 = agree, 4 = strongly agree). For the 7^th^ item in the attitude section, 0 = strongly agree, 1 = agree, 2 = neither agree nor disagree/do not know, 3 = disagree, 4 = strongly disagree. The total attitude score was between 0 and 44. The practice was measured by 7 items using a 5-point Likert scale (0 = strongly disagree or never, 1 = disagree or seldom, 2 = neither agree nor disagree/do not know or sometimes, 3 = agree or often, 4 = strongly agree or always). The total practice score was between 0 and 28.

The questionnaire was distributed to the participants and completed via a professional online survey platform “Wenjuanxing” (www.wjx.cn).^[13]^ Higher scores for each category indicate higher levels of knowledge, attitude, and practice toward stroke and thrombectomy.

## 4. Statistical analysis

Statistical analysis was performed using SPSS 26.0 (IBM, Armonk, NY, USA). The continuous data were expressed as the mean ± standard deviation (SD). Comparisons between 2 groups were conducted using variance analysis or Student *t* test. Categorical data were described with n (%) and compared by the Chi-square test. The correlations among knowledge, attitude, and practice scores were analyzed using Pearson’s correlation analysis. The association of different variables with practice scores was assessed using univariate and multivariate logistic regression analyses. The median practice score of 20 was used as the cutoff value. The relationships between different variables and KAP scores were analyzed using the structural equation model (SEM). A 2-sided *P* value < 0.05 was considered statistically significant.

## 5. Patient and public involvement

No patient involved.

## 6. Results

**Figure 1.**
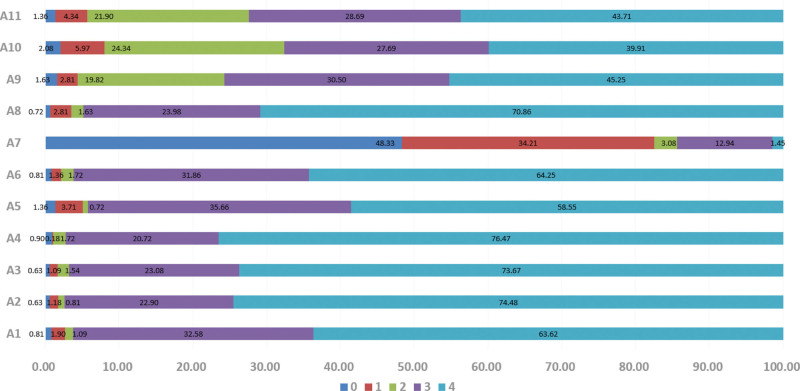
Distribution of attitude scores. “A1–11” were 11 items for practice (part III of Appendix 1, Supplemental Digital Content, http://links.lww.com/MD/N874). “0–4 were scores of each item, and presented in different color.

**Figure 2.**
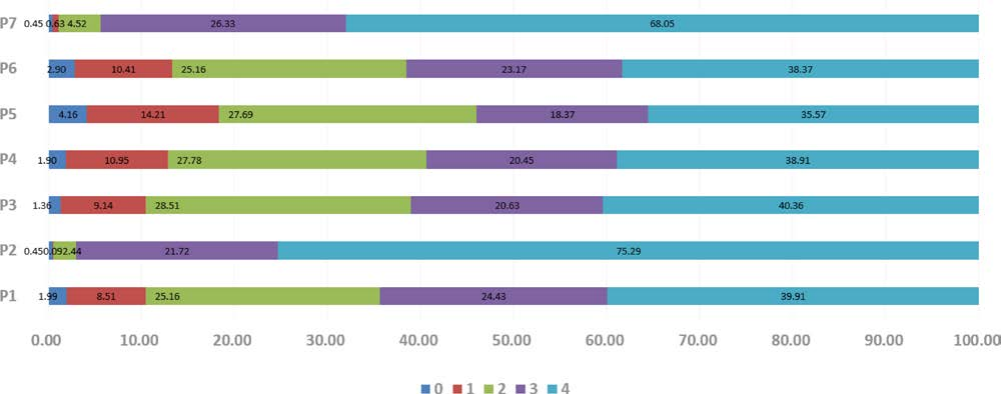
Distribution of practice scores.“P1–7” were 7 items for practice (part IV of Appendix 1, Supplemental Digital Content, http://links.lww.com/MD/N874). “0–4” were scores of each item, and presented in different color.

A total of 1105 medical students participated in the survey, including 754 (68.2%) undergraduate students and 351 (31.8%) graduate students majoring in clinical medicine (48.3%), nursing science (21.4%), medical imaging science (8.5%), traditional Chinese medicine/integration of traditional and Western medicine (14.7%), and other disciplines (7.1%). The mean scores of knowledge, attitude, and practice were 11.1 ± 2.90, 35.37 ± 4.94, and 21.48 ± 5.51 out of 14, 44, and 24, respectively, suggesting adequate knowledge, positive attitude, and proactive practice toward stroke and thrombectomy among the medical students. Additionally, medical students’ age (*P* < .001), education level (*P* < .001), majors (*P* = .004), internship experience in neurosurgery or neurology (*P* < .001), and a family history of stroke (*P* < .001) resulted in significantly different knowledge scores; attitude scores were significantly different in age (*P* = .04), education level (*P* = .022), internship experience in neurosurgery or neurology (*P* < .001), the presence of seniors over 60 years old at home (*P* = .008), and a family history of stroke (*P* < .001); and practice scores varied significantly in age (*P* < .001), sex (*P* < .001), majors (*P* = .010), internship experience in neurosurgery or neurology (*P* < .001), and a family history of stroke (*P* < .001) (Table 1). The distribution of knowledge, attitude, and practice scores were shown in Table 2, Figure 1, and Figure 2, respectively.

**Table 1:** Demographic characteristics of the participants and the scores of knowledge, attitude, and practice.

Variables	N (%)	Knowledge score (mean ± SD)	*P*	Attitude score (mean ± SD)	*P*	Practice score (mean ± SD)	*P*
Total	1105 (100)	11.1 ± 2.90		35.37 ± 4.94		21.48 ± 5.51	
Age (yr)			<.001		.040		<.001
<25	799 (72.3)	10.72 ± 3.14		35.07 ± 5.13		21.09 ± 5.73	
25–30	252 (22.8)	12.12 ± 1.66		36.23 ± 4.37		22.31 ± 4.84	
≥31	54 (4.9)	11.81 ± 2.52		35.74 ± 4.19		23.28 ± 4.34	
Sex			.209		.808		<.001
Male	408 (36.9)	10.95 ± 3.17		35.32 ± 5.78		22.24 ± 5.49	
Female	697 (62.1)	11.18 ± 2.72		35.4 ± 4.38		21.03 ± 5.48	
Education			<.001		.022		.050
Bachelor degree	754 (68.2)	10.74 ± 3.14		35.14 ± 5.12		21.16 ± 5.59	
Master degree or above	351 (31.8)	11.86 ± 2.90		35.87 ± 4.50		22.15 ± 5.27	
Major			.004		.093		.010
Clinical medicine	534 (48.3)	11.18 ± 2.84		35.56 ± 4.89		21.83 ± 5.39	
Nursing science	237 (21.4)	10.92 ± 2.92		34.64 ± 5.31		20.89 ± 5.43	
Medical imaging science	94 (8.5)	11.52 ± 2.48		36.03 ± 3.92		21.72 ± 5.41	
Traditional Chinese medicine/integration of traditional and Western medicine	162 (14.7)	11.41 ± 2.83		35.52 ± 5.16		21.83 ± 5.82	
Others	78 (7.1)	9.85 ± 3.48		35.18 ± 4.58		19.78 ± 5.70	
Internship experience in neurosurgery or neurology			<.001		<.001		<.001
Yes	540 (48.9)	11.93 ± 1.97		36.17 ± 4.17		22.63 ± 4.89	
No	565 (51.1)	10.3 ± 3.38		34.6 ± 5.47		20.38 ± 5.84	
Have seniors over the age of 60 at home			.205		.008		.603
Yes	947 (85.7)	11.15 ± 2.82		35.24 ± 5.08		21.51 ± 5.49	
No	158 (14.3)	10.79 ± 3.31		36.16 ± 3.90		21.27 ± 5.65	
Family history of stroke			<.001		<.001		<.001
Yes	153 (13.8)	11.63 ± 2.24		36.92 ± 4.18		23.57 ± 4.66	
No	793 (71.8)	11.23 ± 2.76		35.23 ± 5.12		21.32 ± 5.54	
Do not know	159 (14.4)	9.89 ± 3.74		34.56 ± 4.37		20.25 ± 5.64	

**Table 2:** Knowledge questions and responses.

No.	Questions	Incorrect response or Don’t know n (%)	Correct response n (%)
K1	Pathogenesis of stroke: a group of disease caused by the sudden rupture or blockage of blood vessel in brain that prevents blood from flowing to the brain and causes the damage to brain tissue.	207 (18.4)	916 (81.6)
K2	Classification of stroke: ischemic stroke, hemorrhagic stroke.	237 (21.1)	886 (78.9)
K3	The appropriate time to initiate intravenous thrombolysis.	903 (80.4)	220 (19.6)
K4	What are the main sources of cerebral emboli?	487 (43.4)	636 (56.6)
K5	Is stroke preventable?	149 (13.3)	974 (86.7)
K6	Early stroke recognition methods include Stroke 120, BEFAST, etc.	252 (22.4)	871 (77.6)
K7	When the early signs and symptoms of stroke were detected, we need to seek medical attention immediately.	90 (8)	1033 (92)
K8	The most common cause of stroke is atherosclerosis.	181 (16.1)	942 (83.9)
K9	Main risk factors of stroke include hypertension, diabetes mellitus, dyslipidemia, smoking, obesity, etc.	108 (9.6)	1015 (90.4)
K10	Early signs and symptoms of stroke include hemiparesis, hemianesthesia, aphasia, dysphonia, vertigo with nausea and vomiting, blurred vision, and unsteadiness in standing and walking, etc.	139 (12.4)	984 (87.6)
K11	Young people are also at the risk of developing stroke, which should be taken seriously.	93 (8.3)	1030 (91.7)
K12	The appropriate time to initiate thrombectomy is within 6 hours.	179 (15.9)	944 (84.1)
K13	Thrombectomy is 1 the most effective procedures to treat stroke.	256 (22.8)	867 (77.2)
K14	The common endovascular thrombectomy procedures for acute ischemic stroke include intra-arterial stent thrombectomy, intra-arterial aspiration, intra-arterial microguidewire fragmentation, intra-arterial thrombolysis, stentoplasty, balloon dilatation plasty, combination of intra-arterial aspiration and stent thrombectomy.	181 (16.1)	942 (83.9)

Furthermore, Pearson’s correlation analysis revealed positive and significant correlations between knowledge-attitude (*R* = 0.397), knowledge-practice (*R* = 0.370), and attitude-practice (*R* = 0.628) (all *P* < .001) (Table 3), reaffirming the relationship among KAP of medical students on stroke and thrombectomy. Multivariate logistic regression analysis showed that adequate knowledge [OR = 1.171 (1.097–1.249), *P* < .001], positive attitude [OR = 1.441 (1.376–1.509), *P* < .001], and male sex [OR = 0.657 (0.481–0.897), *P* = .008] were independently associated with good practice toward stroke and thrombectomy (Table 4).

**Table 3:** Pearson correlation analysis between scores of knowledge, attitude, and practice.

	Knowledge	Attitude	Practice
Knowledge	1		
Attitude	0.397 (*P* < .001)	1	
Practice	0.370 (*P* < .001)	0.628 (*P* < .001)	1

**Table 4:** Univariate and multivariate logistic regression analyses of the association between different variables and the score of practice.

Variables	Univariate logistic regression		Multivariate logistic regression	
	OR (95% CI)	*P*	OR (95% CI)	*P*
Knowledge score	1.294 (1.220–1.371)	<.001	1.171 (1.097–1.249)	<.001
Attitude score	1.464 (1.399–1.532)	<.001	1.441 (1.376–1.509)	<.001
Age (yr)		.001		
<25	REF.			
25–30	1.554 (1.169–2.066)	.002		
≥31	2.148 (1.215–3.797)	.009		
Sex				
Male	REF.		REF	
Female	0.690 (0.540–0.882)	.003	0.657 (0.481–0.897)	.008
Education				
Bachelor degree	REF.			
Master degree or above	1.373 (1.065–1.770)	.015		
Major		.069		
Clinical medicine	REF.			
Nursing science	0.728 (0.535–0.992)	.044		
Medical imaging science	0.973 (0.627–1.508)	.902		
Traditional Chinese medicine/integration of traditional and Western medicine	1.149 (0.808–1.633)	.440		
Others	0.634 (0.390–1.032)	.067		
Internship experience in neurosurgery or neurology				
Yes	REF.			
No	0.541 (0.426–0.687)	<.001		
Have seniors over the age of 60 at home				
Yes	REF.			
No	0.909 (0.648–1.275)	.581		
Family history of stroke		<.001		
Yes	REF.			
No	0.502 (0.351–0.718)	<.001		
Do not know	0.350 (0.221–0.554)	<.001		

Abbreviations: CI = confidence interval, OR = odds ratio, REF = reference.

**Figure 3.**
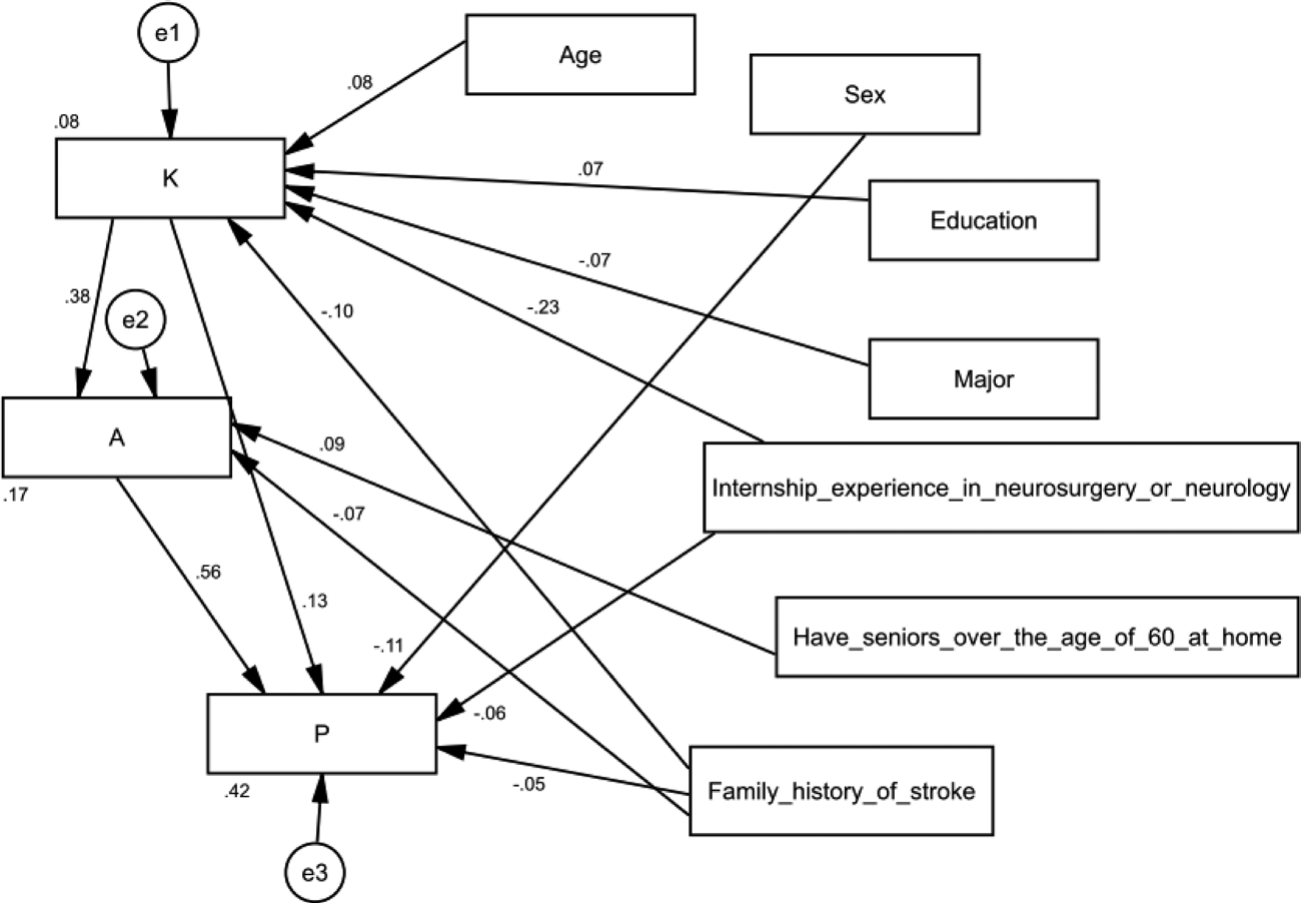
Structural equation model.

SEM revealed that, age (estimate = 0.351, *P* = .005) and education (estimate = 0.370, *P* = .024) positively affected knowledge, while major (estimate = −0.128, *P* = .017), internship experience in neurosurgery or neurology (estimate = −1.321, *P* < .001), and family history of stroke (estimate = −0.557, *P* < .001) negatively influenced knowledge. Knowledge (estimate = 0.649, *P* < .001) and having seniors over the age of 60 at home (estimate = 1.228, *P* = .001) had positive effects on attitudes, while internship experience in neurosurgery or neurology (estimate = −0.471, *P* = .090) and family history of stroke (estimate = −0.596, *P* = .020) had negative impact on attitudes. Moreover, knowledge (estimate = 0.230, *P* < .001) and attitudes (estimate = 0.628, *P* < .001) positively influenced practices, whereas sex (estimate = −1.141, *P* < .001), internship experience in neurosurgery or neurology (estimate = −0.578, *P = *.025), and family history of stroke (estimate = −0.523, *P* = .027) negatively influenced practices (Figure 3 and Table 5).

**Table 5:** Structural equation model.

			Estimate	S.E.	C.R.	*P*	Label
K	<---	Age	0.351	0.124	2.823	.005	
K	<---	Education	0.370	0.164	2.261	.024	
K	<---	Major	−0.128	0.054	−2.387	.017	
K	<---	Internship experience in neurosurgery or neurology	−1.321	0.165	−8.023	<.001	
K	<---	Family history of stroke	−0.557	0.155	−3.599	<.001	
A	<---	Knowledge	0.649	0.049	13.149	<.001	
A	<---	Age	0.034	0.205	0.165	.869	
A	<---	Education	−0.256	0.269	−0.950	.342	
A	<---	Internship experience in neurosurgery or neurology	−0.471	0.278	−1.693	.090	
A	<---	Have seniors over the age of 60 at home	1.228	0.386	3.179	.001	
A	<---	Family history of stroke	−0.596	0.256	−2.330	.020	
P	<---	K	0.230	0.049	4.691	<.001	
P	<---	A	0.628	0.028	22.721	<.001	
P	<---	Age	0.320	0.189	1.693	.090	
P	<---	Sex	−1.141	0.259	−4.413	<.001	
P	<---	Education	−0.107	0.249	−0.431	.667	
P	<---	Major	−0.116	0.081	−1.429	.153	
P	<---	Internship experience in neurosurgery or neurology	−0.578	0.257	−2.248	.025	
P	<---	Family history of stroke	−0.523	0.237	−2.208	.027	

## 7. Discussion

This study suggested that medical students in Henan, China showed adequate knowledge, positive attitude, and proactive practice toward stroke and thrombectomy. Age, sex, education, major, internship experience in neurosurgery or neurology, family history of stroke, having seniors over the age of 60 at home might have impact on their KAP. This study might provide suggestions for talent reserve and education in the field of acute stroke and thrombectomy in China.

In our study, medical students with internship experience in neurosurgery or neurology or a family history of stroke had significantly higher KAP scores than those without experience or family history, suggesting that greater exposure to stroke cases raises the awareness of this disease. Similarly, Das et al have demonstrated that knowledge about stroke was significantly higher among individuals from stroke-affected families.^[14]^ Knowledge regarding the time window for thrombolysis or thrombectomy significantly affects clinical decision-making and hence the rate of stroke-related disability and death.^[15,16]^ However, medical students had the least information about the appropriate time to initiate intravenous thrombolysis as only 19.6% of them rightly answered this question, similar to the study by Pradhan et al showing that only 10.1% of medical students preparing for the undergraduate medical entrance examination know the correct answer.^[17]^ Surprisingly, 84.1% of the medical students knew the optimal time window of thrombectomy, and the correct rates of the other 2 thrombectomy-related knowledge questions were 77.2% and 83.9%, respectively, suggesting that the medical students have good knowledge about thrombectomy.

In this study, over 90% of the participants knew the emergency of stroke, the risk factors of stroke, as well as the occurrence of stroke in young adults. Consistently, most of the samples in other studies are aware of the importance of seeking emergency care as early as possible after a stroke is identified.^[18,19]^ In agreement with our data, Malaeb et al have found that <10% of the Lebanese population considered stroke an old person disease.^[18]^ We did not observe significant gender differences in risk factors awareness, consistent with previous reports.^[20,21]^ However, some studies have shown that females had better knowledge about stroke risk factors than males,^[18,22–24]^ whereas other studies have demonstrated better knowledge among men.^[25,26]^ The discrepancy is possibly due to the differences in sex distribution, access to education, and information availability in different countries.^[27]^ In addition, our results revealed that graduate students were more knowledgeable about stroke and thrombectomy than undergraduate students. Similar results were observed between medical science-related majors and other majors. Studies have suggested that having a higher education level is associated with a greater degree of knowledge of stroke,^[28,29]^ which is in agreement with our results.

For the attitude, we found that participants aged 25 to 30 years old, studying in graduate school, with internship experience in neurosurgery or neurology, and having a family member over 60 years old or a family history of stroke had a more positive attitude toward stroke and thrombectomy than their counterparts. The improved attitude may result from higher education level and greater exposure to stroke cases. However, Das et al have demonstrated that stroke-affected families and non-stroke-affected families were comparable regarding the attitude toward stroke,^[14]^ possibly due to bias in the participants, questionnaire, and geometry features.

We noticed that higher knowledge and attitude scores were generally accompanied by increased practice scores, except for when sex was considered. Male students performed better than female students in the practice category, despite the comparable knowledge scores and attitude scores between the 2 groups. Similar sex difference in practice has been observed in a KAP study on cardiovascular disease.^[30]^ Moreover, multivariate linear regression analysis revealed that sex, knowledge, and attitude were independent predictors of practice. Thus, sex-specific strategies in medical education about stroke and thrombectomy may address the sex difference in practice.

The study used an SEM model to analyze the relationship between variables and the KAP scores.^[31]^ The findings highlight the importance of age, education, internship experience, family history, and sex in shaping knowledge, attitudes, and practices related to stroke and thrombectomy among medical students. These findings align with previous research that highlights the positive impact of integrating stroke prevention education into the early years of medical curriculum.^[32]^ Furthermore, a study focusing on nursing students, many of whom had personal experiences through family history and caregiving, demonstrated a commendable level of knowledge about the condition. This suggests that the students’ solid foundation in stroke knowledge can be attributed to their personal connections and practical involvement in stroke care.^[33]^ Thus, the findings of this study emphasize the importance of incorporating stroke prevention education, considering influential factors, and harnessing personal experiences to enhance medical students’ KAP regarding stroke and thrombectomy.

This study has limitations. Firstly, data were gathered by self-reporting, which might be relatively unreliable due to self-reporting bias when compared with other sources of information such as medical records and laboratory measurements. In addition, this study was conducted among medical students who are more knowledgeable about stroke and thrombectomy than the general population. Therefore, the results do not represent the KAP toward stroke and thrombectomy in the community. Moreover, participants were recruited from medical schools in Henan, China. Further studies need to be conducted in more areas with larger sample sizes to better understand the KAP of stroke in the whole country.

## 8. Conclusions

In conclusion, this is the first study to evaluate the KAP of stroke and thrombectomy in medical students in Henan, China, who showed adequate knowledge, positive attitude, and proactive practice toward stroke and thrombectomy. Age, sex, education, major, internship experience in neurosurgery or neurology, family history of stroke, having seniors over the age of 60 at home might have impact on their KAP. Nevertheless, more efforts are still needed to increase the knowledge about the time window of thrombolysis and to improve the practice of female medical students.

## Author contributions

**Conceptualization:** Jiabin Wang.

**Data curation:** Lingfeng Shu, Qinghai Dai, Tao Wu.

**Formal analysis:** Jiabin Wang.

**Writing – review & editing:** Haobo Gao, Pengcheng Zhu, Hongtu Tan.

**Writing – original draft:** Lingfeng Shu, Qinghai Dai, Tao Wu.

## Supplementary Material


