# Supplementary material for: Knowledge, attitude, and practice of stroke and thrombectomy among medical students in Henan, China
Source: Medicine (Baltimore). 2024 Nov 8;103(45):e40441. doi: 10.1097/MD.0000000000040441 (PMC11557070; doi:10.1097/MD.0000000000040441)
Supplement: Supplementary file 1 [file medi-103-e40441-s001.docx]

**Appendix**

| Dear medical students.  Hello!  We are researchers from XXX Hospital and we invite you to participate in our research! In order to learn about the professionalism of medical students and to targeted strengthening education in this area. This research needs to collect some data to investigate Knowledge, attitude, and practice of acute stroke and thrombectomy among medical students  To participate in our research, you only need to complete this questionnaire according to your actual situation. The questionnaire is confidential without any disclosion of your information, which can be rest assured.  Thank you for taking the time out of your busy schedule to support our scientific research!  □ I am aware of and agree to the use of the collected data for scientific research. |
| --- |

| **Part I Basic Information** | |
| --- | --- |
| 1. Your age: | a. < 25  b. 25-30  c. 30-35  d. ≥ 35 |
| 2. Your sex: | a. Male  b. Female |
| 3. Your education: | a. Undergraduate  b. Graduate  c. PhD |
| 4. Your major: . | |
| 5. Do you have any Internship experience in neurosurgery or neurology? | a. Yes  b. No |
| 5.1. If yes, is the internship hospital a teaching hospital? | a. Yes  b. No |
| 6. Do you have seniors over the age of 60 at home? | a. Yes  b. No |
| 7. Do you have a family history of stroke? | a. Yes  b. No  c. Do not know |
| 7.1. If yes, whether this member has undergone thrombectomy? | a. Yes  b. No  c. Do not know |

| **Part II Knowledge on acute stroke and thrombectomy** | |
| --- | --- |
| K1. Pathogenesis of stroke: a group of disease caused by the sudden rupture or blockage of blood vessel in brain that prevents blood from flowing to the brain and causes the damage to brain tissue.  a. Correct b. Incorrect c. Don’t know | |
| K2. Classification of stroke: ischemic stroke, hemorrhagic stroke.  a. Correct b. Incorrect c. Don’t know | |
| K3. The appropriate time to initiate intravenous thrombolysis.  a. Within 3 hours after disease onset b. Within 4.5 hours after disease onset c. Within 6 hours after disease onset d. Within 12 hours after disease onset | |
| K4. What are the main sources of cerebral emboli?  a. Cardiac embolism b. Air embolism c. Fat embolism d. Cerebral arteriosclerosis embolism | |
| K5. Is stroke preventable?  a. Correct b. Incorrect c. Don’t know |  |
| K6. Early stroke recognition methods include Stroke 120, BEFAST, etc.  a. Correct b. Incorrect c. Don’t know |  |
| K7. When the early signs and symptoms of stroke were detected, we need to seek medical attention immediately.  a. Correct b. Incorrect c. Don’t know |  |
| K8. The most common cause of stroke is atherosclerosis.  a. Correct b. Incorrect c. Don’t know |  |
| K9. Main risk factors of stroke include hypertension, diabetes mellitus, dyslipidemia, smoking, obesity, etc.  a. Correct b. Incorrect c. Don’t know |  |
| K10. Early signs and symptoms of stroke include hemiparesis, hemianesthesia, aphasia, dysphonia, vertigo with nausea and vomiting, blurred vision, and unsteadiness in standing and walking, etc.  a. Correct b. Incorrect c. Don’t know |  |
| K11. Young people are also at the risk of developing stroke, which should be taken seriously.  a. Correct b. Incorrect c. Don’t know |  |
| K12. The appropriate time to initiate thrombectomy is within 6 hours.  a. Correct b. Incorrect c. Don’t know |  |
| K13. Thrombectomy is one the most effective procedures to treat stroke.  a. Correct b. Incorrect c. Don’t know |  |
| K14. The common endovascular thrombectomy procedures for acute ischemic stroke include intra-arterial stent thrombectomy, intra-arterial aspiration, intra-arterial microguidewire fragmentation, intra-arterial thrombolysis, stentoplasty, balloon dilatation plasty, combination of intra-arterial aspiration and stent thrombectomy.  a. Correct b. Incorrect c. Don’t know |  |

| **Part III Attitude on acute stroke and thrombectomy** | |
| --- | --- |
| A1. I believe that strokes can be prevented.  a. Strongly Agree; b. Agree; c. Neutral; d. Disagree; e. Strongly disagree | |
| A2. I believe that stroke should be diagnosed aggressively and treated appropriately at an early stage, only then can we back to the health without stroke.  a. Strongly Agree; b. Agree; c. Neutral; d. Disagree; e. Strongly disagree | |
| A3. I think everyone should be aware of stroke first aid pathways and methods.  a. Strongly Agree; b. Agree; c. Neutral; d. Disagree; e. Strongly disagree | |
| A4. I believe that patients and their families should also be aware of the conventional treatment of stroke.  a. Strongly Agree; b. Agree; c. Neutral; d. Disagree; e. Strongly disagree | |
| A5. I believe that most stroke patients will have varying degrees of residual symptoms that affect their work and life.  a. Strongly Agree; b. Agree; c. Neutral; d. Disagree; e. Strongly disagree | |
| A6. I believe that thrombectomy is meaningful in improving the long-term quality of life of patients.  a. Strongly Agree; b. Agree; c. Neutral; d. Disagree; e. Strongly disagree | |
| A7. I believe that most stroke patients should be treated with thrombectomy.  a. Strongly Agree; b. Agree; c. Neutral; d. Disagree; e. Strongly disagree | |
| A8. I think doctors need to explain in detail the rationale, benefits, and risks of thrombectomy to the patient's family before starting the treatment, even if it may takes a long time.  a. Strongly Agree; b. Agree; c. Neutral; d. Disagree; e. Strongly disagree | |
| A9. I am willing to work in the field of stroke prevention and treatment.  a. Strongly Agree; b. Agree; c. Neutral; d. Disagree; e. Strongly disagree | |
| A10. I am willing to work in neurological and cerebrovascular intervention without the fear of X-rays.  a. Strongly Agree; b. Agree; c. Neutral; d. Disagree; e. Strongly disagree | |
| A11. I am willing to engage in fundamental experimental work on the development and improvement of the devices related to cerebrovascular thrombectomy and aspiration.  a. Strongly Agree; b. Agree; c. Neutral; d. Disagree; e. Strongly disagree | |
| **Part IV Practice on acute stroke and thrombectomy** |  |
| P1. I will follow and participate in stroke health propaganda.  a. Always; b. Often; c. Occasionally; d. Seldom; e. Never |  |
| P2. Once I find a patient with stroke, I will promptly call the emergency number 120.  a. Strongly Agree; b. Agree; c. Neutral; d. Disagree; e. Strongly disagree |  |
| P3. I will take the initiative to learn about stroke and retrieval of thrombectomy.  a. Always; b. Often; c. Occasionally; d. Seldom; e. Never |  |
| P4. I will take the initiative to learn about post-thrombolysis care.  a. Always; b. Often; c. Occasionally; d. Seldom; e. Never |  |
| P5. I will take the initiative to attend training in specialty care and innovative practice of stroke.  a. Always; b. Often; c. Occasionally; d. Seldom; e. Never |  |
| P6. I will educate my relatives and friends about what I have learned about stroke and thrombectomy.  a. Always; b. Often; c. Occasionally; d. Seldom; e. Never |  |
| P7. When my friends or relatives ask me for advice about the decision of thrombectomy therapy, I will suggest them to follow the treatment plan, which was evaluated and recommended by the professional.  a. Strongly Agree; b. Agree; c. Neutral; d. Disagree; e. Strongly disagree |  |
